# Supplementary figures and images for: Effects of parental care on skin microbial community composition in poison frogs
Source: eLife. 2025 Jul 31;14:RP103331. doi: 10.7554/eLife.103331 (PMC12313234; doi:10.7554/eLife.103331)

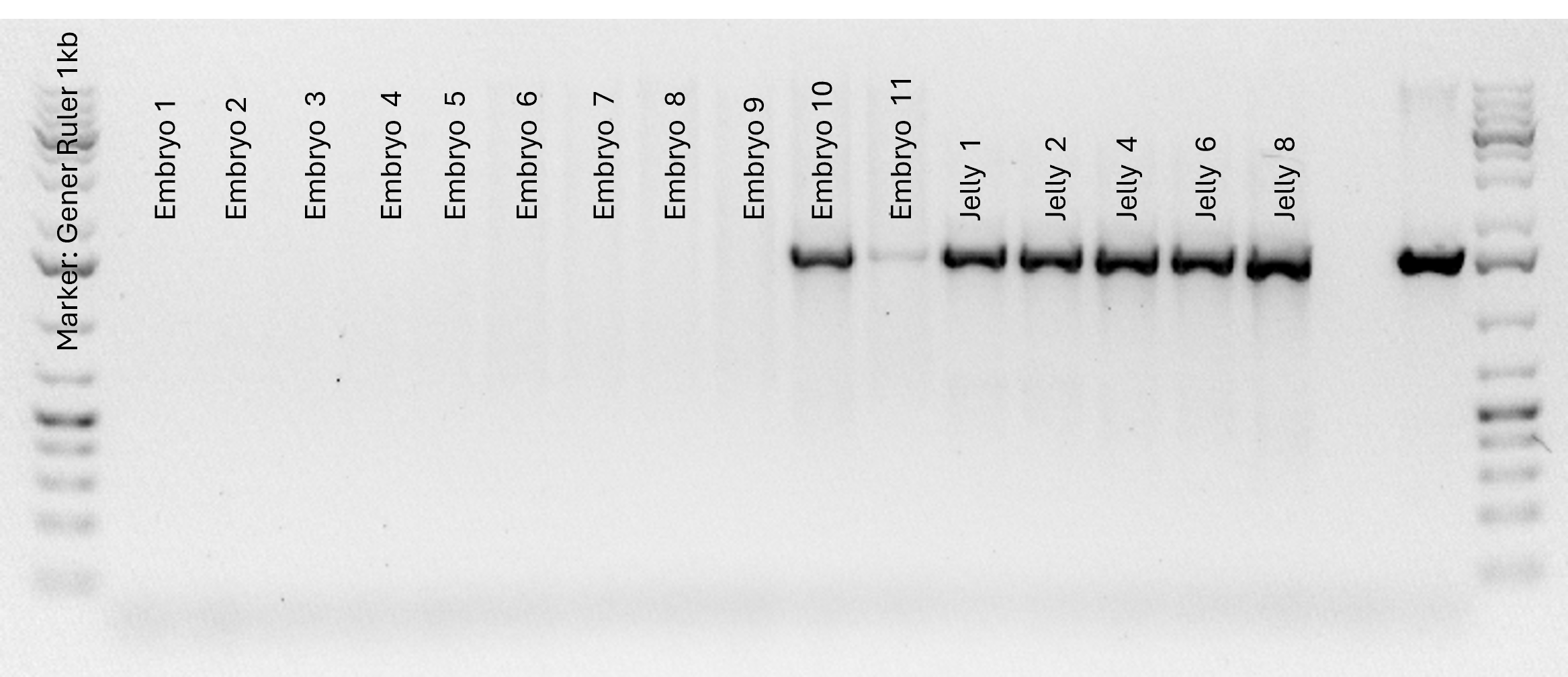

Supplement: Figure 1—figure supplement 1—source data 1. [file elife-103331-fig1-figsupp1-data1.zip › Figure 1- supplementary figure 1-source data 1-inverted, labelled/Figure 1- supplementary figure 1 B_upper row.png]

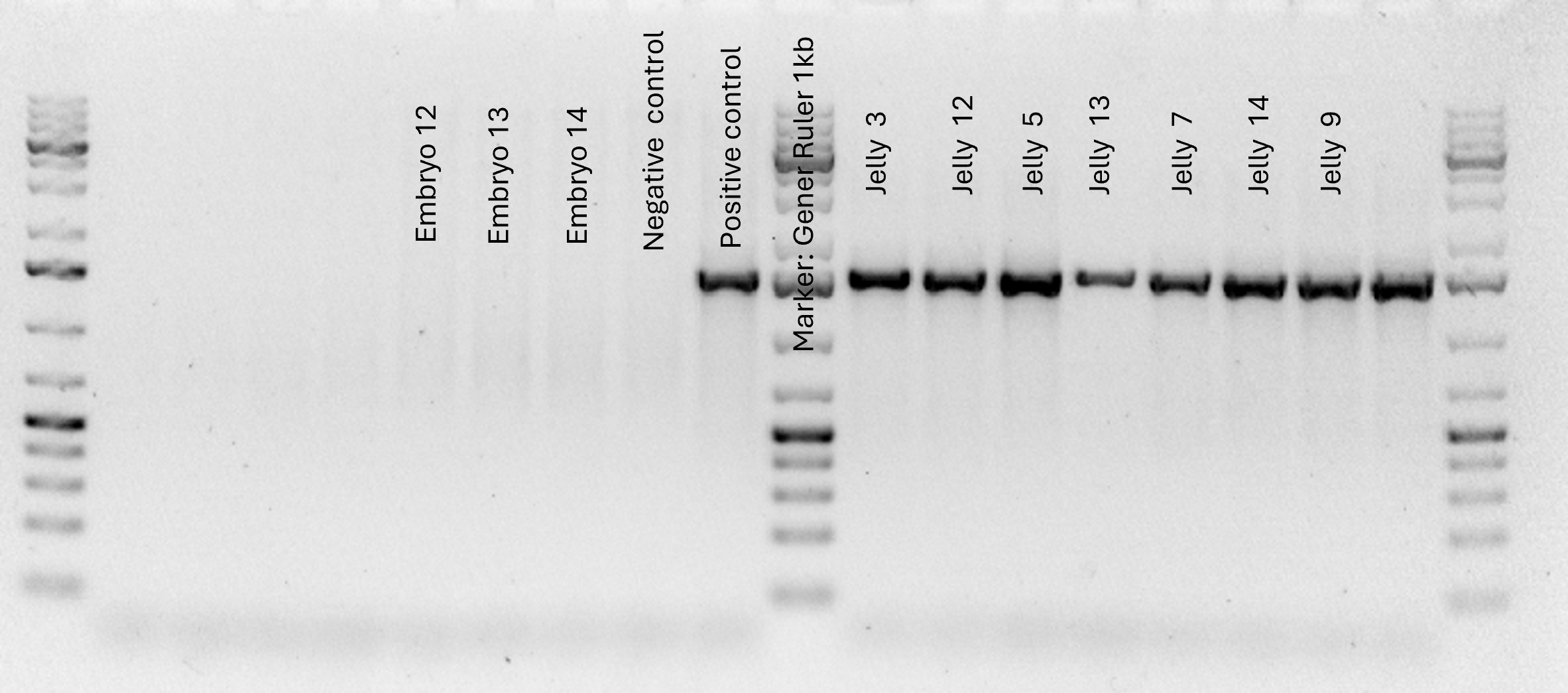

Supplement: Figure 1—figure supplement 1—source data 1. [file elife-103331-fig1-figsupp1-data1.zip › Figure 1- supplementary figure 1-source data 1-inverted, labelled/Figure 1- supplementary figure 1 B_lower row.png]

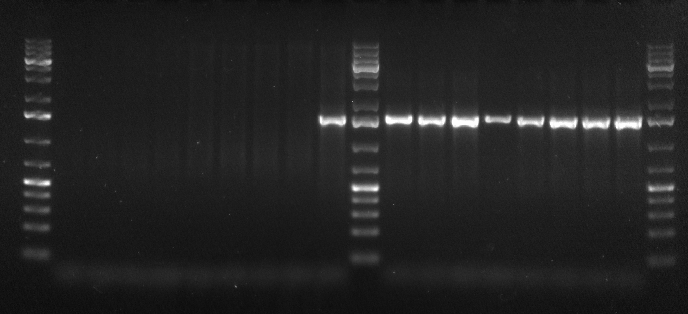

Supplement: Figure 1—figure supplement 1—source data 2. [file elife-103331-fig1-figsupp1-data2.zip › Figure 1- supplementary figure 1-source data 1 - unedited/Figure 1- supplementary figure 1-source data 1_lower row.PNG]

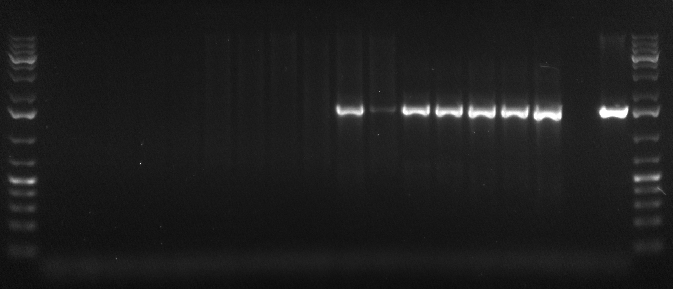

Supplement: Figure 1—figure supplement 1—source data 2. [file elife-103331-fig1-figsupp1-data2.zip › Figure 1- supplementary figure 1-source data 1 - unedited/Figure 1- supplementary figure 1-source data 1_upper row_raw.PNG]
