# Supplementary material for: Effects of parental care on skin microbial community composition in poison frogs
Source: eLife. 2025 Jul 31;14:RP103331. doi: 10.7554/eLife.103331 (PMC12313234; doi:10.7554/eLife.103331)
Supplement: Supplementary file 1. — (a) 16S rRNA gene copy number variations in embryos and jelly measured by ddPCR. (b) Source proportions of Os and Rv communities in the microbiome of tadpoles transported by Os, and their sibling transported by Rv or non-transported. (c) Taxonomic composition of microbiome samples from wild tadpoles and adults. (d) Statistical analysis of alpha diversity measures across species and life stages and environments. (e) PERMANOVA results for Principal Coordinate-located in the nature reserve ‘Les Analysis on Bray Curtis distances. (f) Identity and number of core taxa across abundance and prevalence cutoffs. (h–j) Shared ASVs between transported tadpoles and their caregivers, including relative and scaled absolute abundances and presence in non-transported tadpoles. [file elife-103331-supp1.docx]

**Supplementary File 1**

Effects of parental care on skin microbial community composition in poison frogs

Marie-Therese Fischer, Katherine S. Xue, Elizabeth K. Costello, Anna Robaczewska, Mai Dvorak, Gaëlle Raboisson, Stephanie N. Caty, David A. Relman, Lauren A. O’Connell

**Supplementary File 1a: Copy number variations of the 16S rRNA gene between embryos and jelly detected by ddPCR.** DNA extracted from jellies, hatched and unhatched embryos (N = 14 eggs) was analyzed using a QX200 AutoDG Droplet Digital PCR system (Bio-Rad) with universal 16S rRNA primers (331F/797R) and 16S rRNA FAM probes. Displayed copy numbers were corrected for dilution factor, extraction elution volume, and sample ddPCR volume and represent 16S rRNA copy numbers/µl present in the jelly or embryo of one Rv egg. Mean copy numbers/µl and standard deviations were calculated for each Gosner stage.

| **ID** | **group** | **copies/µl** | **mean copies/µl/stage ± STD** | **Gosner stage** |
| --- | --- | --- | --- | --- |
| Embryo 1 | unhatched | 3463 | 3463 (~3x10^3^) | st 10 |
| Embryo 2 | unhatched | 2161 |  | st 18 |
| Embryo 3 | unhatched | 999 | 1611 (~1 x 10^3^) **±** 583 | st 18 |
| Embryo 12 | unhatched | 1673 |  | st 18 |
| Embryo 4 | unhatched | 3853 |  | st 20 |
| Embryo 5 | unhatched | 766 | 1896 (~2 x 10^3^) **±** 1702 | st 20 |
| Embryo 13 | unhatched | 1068 |  | st 20 |
| Embryo 6 | unhatched | 1886 |  | st 23 |
| Embryo 7 | unhatched | 742 | 4737 (~5 x 10^3^) **±** 5957 | st 23 |
| Embryo 14 | unhatched | 11584 |  | st 23 |
| Embryo 8 | unhatched | 5561 | 3554 (~4 x 10^3^) **±** 2837 | st 24 |
| Embryo 9 | unhatched | 1548 |  | st 24 |
| Embryo 10 | hatched | 48159335 | 24448337 (~2 x 10^7^) **±** 33532414 | st 24 |
| Embryo 11 | hatched | 737340 |  | st 24 |
| Jelly 1 | jelly | 37998434 | 18029604 (~2 x 10^7^) **±** 583 | st 10 |
| Jelly 2 | jelly | 3550824 |  | st 18 |
| Jelly 3 | jelly | 4235222 |  | st 18 |
| Jelly 12 | jelly | 21107921 |  | st 18 |
| Jelly 4 | jelly | 21990138 |  | st 20 |
| Jelly 5 | jelly | 457417 |  | st 20 |
| Jelly 13 | jelly | 19812023 |  | st 20 |
| Jelly 6 | jelly | 8318251 |  | st 23 |
| Jelly 7 | jelly | 18013704 |  | st 23 |
| Jelly 14 | jelly | 11299376 |  | st 23 |
| Jelly 8 | jelly | 66115451 |  | st 24 |
| Jelly 9 | jelly | 3456484 |  | st 24 |

**Supplementary File 1b: Source proportions of Os and Rv communities** **in the microbiome of tadpoles transported by Os, and their sibling transported by Rv or non-transported.** Siblings belonging to the same clutch are indicated by color blocks. Abbreviations: exp group = experimental group, syl = transported by Os (N = 9), var = transported by Rv (N = 7), control = non-transported (N = 6), transp = transported.

| **Tadpole ID** | **Exp group** | **Family ID** | **% Os** | **% Rv** | **% more Os than in Rv-transp sibling** | **% more Os than in non-transp sibling** |
| --- | --- | --- | --- | --- | --- | --- |
| 1 | control | 2 | 1.759 | 16.434 |  |  |
| 2 | syl | 2 | 41.163 | 6.570 | 39.33 | 39.44 |
| 3 | syl | 2 | 70.311 | 1.088 | 68.478 | 68.552 |
| 4 | var | 2 | 1.833 | 2.640 |  |  |
| 5 | control | 3 | 0.000 | 2.132 |  |  |
| 6 | syl | 3 | 72.973 | 1.351 | 4.27 | 72.973 |
| 7 | var | 3 | 32.766 | 18.816 |  |  |
| 8 | control | 4 | 0.175 | 0.526 |  |  |
| 9 | syl | 4 | 68.349 | 0.272 | 57.965  59.978 | 68.174  9.7 |
| 10 | var | 4 | 8.371 | 17.400 |  |  |
| 11 | var | 4 | 10.385 | 68.956 |  |  |
| 12 | control | 5 | 0.000 | 0.900 |  |  |
| 13 | syl | 5 | 0.874 | 0.291 | 0.872 | 0.874 |
| 14 | syl | 5 | 34.730 | 0.811 | 34.728 | 34.73 |
| 15 | var | 5 | 0.002 | 9.623 |  |  |
| 16 | control | 6 | 0.250 | 0.375 |  |  |
| 17 | syl | 6 | 50.586 | 0.554 | 49.876 | 5.586 |
| 18 | var | 6 | 0.710 | 0.118 |  |  |
| 19 | control | 7 | 0.000 | 0.678 |  |  |
| 20 | syl | 7 | 22.593 | 2.222 | 22.534 | 22.593 |
| 21 | syl | 7 | 9.381 | 0.442 | 7.158 | 9.381 |
| 22 | var | 7 | 0.059 | 43.985 |  |  |

**Supplementary File 1c: Taxonomic composition of microbiome samples from wild tadpoles and adults.** Total number of phyla and families encountered in each group were calculated from unrarefied data, averaged group values and standard deviations were calculated from a rarefied dataset. Batrachochytrium dendrobatidis (Bd) positive samples were extracted from an ITS sequencing dataset and confirmed with a nested PCR targeting Bd. Abbreviations: #: number, Bd +: number of samples positive for Batrachochytrium dendrobatidis, avg: average, inhibit.: inhibiting. Maximas are displayed bold, lowest number of Bd inhibiting taxa are marked grey.

| **Species** | **Sample** | **N** | **Total # Phyla** | | **Avg # Phyla** | **Total # Families** | **Avg # Families** | **Bd +** | **Avg # Bd inhibit. taxa** |
| --- | --- | --- | --- | --- | --- | --- | --- | --- | --- |
|  | A | 44 | | 32 | 7.3 ± 3.9 | 265 | 26.4 ± 19.1 | 2 (4.5 %) | 5.7 ± 3.6 |
| ***R. variabilis*** | T | 21 | | 25 | 9.1 ± 4.1 | 185 | 29.1 ± 16.2 | 1 (4.7 %) | 1.1 ± 0.5 |
|  | W | 21 | | **32** | **17.5** ± 3.2 | **283** | **73.7 ± 14.6** | - | 1.6 ± 1.6 |
|  | A | 10 | | 28 | 9.2 ± 3.5 | 208 | 34.7 ± 19.8 | 1 (10%) | 7.6 ± 6.0 |
| ***A. femoralis*** | T | 8 | | 21 | 7.3 ± 2.5 | 104 | 29 ± 13.6 | 0 | 3.1 ± 2.0 |
|  | W | 3 | | **33** | **14.3** ± 1.5 | **267** | **75.3 ± 2.1** | - | 11.7 ± 4.0 |
|  | A | 10 | | 16 | 9.7 ± 2.9 | 112 | 31.9 ± 11.6 | 0 | 6.3 ± 4.4 |
| ***L. longirostris*** | T | 14 | | 19 | 4.3 ± 2.0 | 98 | 11.6 ± 6.0 | 0 | 0.4 ± 0.5 |
|  | W | 6 | | **25** | **13.8** ± 1.3 | **172** | **66.5 ± 5.4** | - | 3.2 ± 1.2 |

**Supplementary File 1d: Statistical analysis of alpha diversity measures across species and life stages and environments.** Statistical tests for alpha diversity were performed on rarefied datasets. Significant ANOVAs were followed by Tukey post-hoc tests. Sample sizes for each comparison are provided under the respective group. Significance levels of adjusted p values are indicated by * (p < 0.05), ** (p < 0.01) or *** (p < 0.001). Abbreviations: T: tadpole, A: adult, Rv: Ranitomeya variabilis, Af: Allobates femoralis, Ll: Leptodactylus longirostris.

| **Species** | **Comparison** | **Diversity measure** | **Test type** | **Df** | **Test Statistic** | **p.adj** |
| --- | --- | --- | --- | --- | --- | --- |
| *Rv* | A - T | Observed ASV | KW | 1 | Chi^2^ = 4.7004 | 0.0905 |
|  | (44 – 21) | Shannon | ANOVA | 1 | F = 0.5267 | 1.4121 |
|  |  | evenness | ANOVA | 1 | F = 0.0099 | 2.7636 |
| *Af* | A -T | Observed ASV | KW | 1 | Chi^2^ = 0.19737 | 1.9707 |
|  | (10 – 8) | Shannon | ANOVA | 1 | F = 0.1843 | 2.0202 |
|  |  | evenness | KW | 1 | Chi^2^ = 0.95526 | 0.9852 |
| *Ll* | A – T | Observed ASV | KW | 1 | Chi^2^ = 7.2612 | 0.0211* |
|  | (10 – 10) | Shannon | ANOVA | 1 | F = 31.143 | < 0.001*** |
|  |  | evenness | ANOVA | 1 | F = 33.492 | < 0.001*** |
| *Rv – Ll* | T - T | Observed ASV | KW | 2 | Chi^2^ = 10.158 | 0.0127* |
|  | (21 – 10) | Shannon | ANOVA | 2 | F = 10.973 | 0.0020** |
|  |  | evenness | ANOVA | 2 | F = 10.982 | 0.0024** |
| *Af – Ll* | T - T | Observed ASV | KW | 2 | Chi^2^ = 10.158 | 0.0149* |
|  | (8 – 10) | Shannon | ANOVA | 2 | F = 10.973 | 0.0003** |
|  |  | evenness | ANOVA | 2 | F = 10.982 | 0.0003** |
| Rv - Ll | W – W  (21 – 6) | Observed ASV  Shannon  evenness | ANOVA  ANOVA  ANOVA | 2  2  2 | F = 18.752  F = 11.205  F = 0.01104 | 0.0037*  0.0023*  0.0212* |
| Rv- Af | W – W  (21 – 3) | Observed ASV  Shannon  evenness | ANOVA  ANOVA  ANOVA | 2  2  2 | F = 18.752  F = 11.205  F = 0.01104 | < 0.001***  0.005*  0.1278 |
| Af - Ll | W – W  (3-6) | Observed ASV  Shannon  evenness | ANOVA  ANOVA  ANOVA | 2  2  2 | F = 18.752  F = 11.205  F = 0.01104 | < 0.001***  0.9936  0.8436 |

**Supplementary File 1e: PERMANOVA for Principal Coordinate Analysis on Bray Curtis distances**. Adonis permutation was followed by a pairwise adonis to determine if communities of groups cluster distinctly. Sample sizes as indicated in Supplementary File 1d. Significance levels of p and adjusted p values are indicated by * (p < 0.05), ** (p < 0.01) or *** (p < 0.001).

| **Comparison** | **Df** | **SumsOfSqs** | **F Model** | **R2** | **P value** | **Adj. p** |
| --- | --- | --- | --- | --- | --- | --- |
| *A_Rv* vs *A_Ll* | 1 | 1.992 | 7.796 | 0.130 | 0.001 *** | 0.036 * |
| *A_Rv* vs *A_Af* | 1 | 3.158 | 11.513 | 0.181 | 0.001 *** | 0.036 * |
| *A_Ll* vs *A_Af* | 1 | 2.081 | 7.941 | 0.306 | 0.001 *** | 0.036 * |
| *A_Rv* vs *T_Rv* | 1 | 6.659 | 27.721 | 0.306 | 0.001 *** | 0.036 * |
| *T_Ll* vs *A_Ll* | 1 | 4.089 | 29.143 | 0.570 | 0.001 *** | 0.036 * |
| *T_Af* vs *A_Af* | 1 | 2.338 | 9.503 | 0.373 | 0.001 *** | 0.036 * |
| *T_Ll* vs *T_Af* | 1 | 3.711 | 32.184 | 0.617 | 0.001 *** | 0.036 * |
| *T_Af* vs *T_Rv* | 1 | 3.671 | 20.619 | 0.433 | 0.001 *** | 0.036 * |
| *T_Ll* vs *T_Rv* | 1 | 5.779 | 38.646 | 0.539 | 0.001 *** | 0.036 * |
| *T_Af* vs *Af_water* | 1 | 0.842 | 6.322 | 0.413 | 0.013 * | 0.468 |
| *T_Rv* vs *Rv_water* | 1 | 4.704 | 26.064 | 0.389 | 0.001 *** | 0.036 * |
| *T_Ll* vs *Ll_water* | 1 | 3.221 | 32.362 | 0.643 | 0.001 *** | 0.036 * |

**Supplementary File 1f: Number of core taxa (genus level agglomerated) across different prevalence and abundance cutoffs.** Sample sizes as indicated in Supplementary File 1d. Abbreviations: prev = prevalence, abd = abundance, aqu env = aquatic environment.

| **Sample** | **prev 100% (no abd)** | **prev 75%, abd 1%** | **prev 75%, abd 0.1%** | **prev 75% (no abd)** |
| --- | --- | --- | --- | --- |
| ***Rv* adults** | 0 | 1 | 1 | 2 |
| ***Rv* tadpoles** | 0 | 3 | 6 | 14 |
| ***Rv* aqu env** | 0 | 4 | 24 | 69 |
| ***Af* adults** | 0 | 0 | 4 | 7 |
| ***Af* tadpoles** | 0 | 7 | 7 | 7 |
| ***Af* aqu env** | 0 | 12 | 81 | 236 |
| ***Ll* adults** | 0 | 2 | 4 | 4 |
| ***Ll* tadpoles** | 0 | 2 | 2 | 6 |
| ***Ll* aqu env** | 0 | 6 | 46 | 110 |

**Supplementary File 1g: Identity of core genera across different occurrence and abundance cutoffs**. Prevalence of each genus in the aquatic environment of the respective tadpoles is indicated by "N" (no) or "Y" (yes). Colors indicate cutoff levels (yellow: prevalence > 75%, blue: prevalence > 75% and abundance > 0.1%, black: prevalence > 75% and abundance > 1%), H_2_O = aquatic environment. Sample sizes as indicated in Supplementary File 1d.

| **Sample** | **Core Genera** | **H_2_O** |
| --- | --- | --- |
| ***Rv* adults** | **"Rosenbergiella"**  "Methylobacterium-Methylorubrum" | **N**  N |
| ***Rv* tadpoles** | **"Cetobacterium"**  **"Novosphingobium"**  **"Pelomonas"**  **"Bacteroides"**  **"Alistipes"**  **"Bacteria_Verrucomicrobiota_Verrucomicrobiae_Opitutales_Puniceicoccaceae "**  "Rikenella"  "Desulfovibrio"  "Clostridium_sensu_stricto_1"  "Tyzzerella"  "Monoglobus"  "Ruminococcus"  "Roseiarcus"  "Rhodomicrobium" | **Y**  **Y**  **Y**  **N**  **N**  **N**  N  Y  Y  Y  N  N  Y  Y |
| ***Af* adult** | **"Candidatus_Hemobacterium"**  **"Pedobacter"**  **"Pseudomonas"**  **"Allorhizobium-Neorhizobium-Pararhizobium-Rhizobium"**  "Bacteria_Planctomycetota_Planctomycetes_Gemmatales_Gemmataceae_uncultured"  "Bradyrhizobium"  "Burkholderia-Caballeronia-Paraburkholderia" | **N**  **N**  **Y**  **N**  Y  Y  **Y** |
| ***Af* tadpoles** | **"Mycobacterium"**  **"Bacteria_Bacteroidota_Bacteroidia_Bacteroidales_Barnesiellaceae "**  **"Rikenella"**  **"Dinghuibacter"**  **"Dechloromonas"**  **"Denitratisoma"**  **"Aeromonas"** | **Y**  **Y**  **Y**  **Y**  **Y**  **Y**  Y |
| ***Ll* adults** | **"Methylobacterium-Methylorubrum"**  **"Rhodomicrobium"**  **"Burkholderia-Caballeronia-Paraburkholderia"**  **"Candidatus_Xiphinematobacter"** | **Y**  **Y**  **Y**  **Y** |
| ***Ll* tadpoles** | **"Pandoraea"**  **"Curvibacter"**  "Desulfovibrio"  "Tyzzerella"  "Aquitalea"  "Rikenella" | **Y**  **Y**  Y  Y  Y  N |

**Supplementary File 1h: Identity, relative contribution of the caregiver’s microbiome, relative abundance in tadpole communities, and source-pool presence of ASVs shared between transported tadpoles and their adult caregivers.** Relative contribution of the caregiver’s microbiome was determined with Sourcetracker. Abundances over 10% are indicated bold. Abbreviations: T = tadpole, A = adult, relA = relative abundance.

| **tadpole ID** | **Proportion of caregiver microbiome (Sourcetracker)** | **ASVs shared T-A** | **Presence in source: adult (A), adult or water (A/W)** | **ASV** | **relA (%)** | **Presence in caregivers 10 most abundant ASV** |
| --- | --- | --- | --- | --- | --- | --- |
| **transported 1** | 1% | 2 | A  A/W | 102:Akkermansia, 144:Enterobacteriaceae | 1.847  1.450 | yes  no |
| **transported 2** | 17% | 1 | A/W | 144:Enterobacteriaceae | **19.258** | yes |
| **transported 3** | 0 % | 1 | A/W | 312:Elsteraceae | 0.125 | no |
| **transported 4** | 0 % | 0 | 0 | - | 0 | - |
| **transported 5** | 0 % | 0 | 0 | - | 0 | - |
| **transported 6** | 15 % | 2 | A  A/W | 138:Aquitalea, 421:Methylocella | 0.842  **18.050** | yes  yes |
| **transported 7** | 0% | 1 | A/W | 88:Methylobacterium-Methylorubrum | 0.020 | yes |
| **transported 8** | 9% | 1 | A | 911:Staphylococcus | **10.329** | yes |
| **transported 9** | 0% | 2 | A  A/W | 28:Akkermansia, 3092:Pseudomonas | 1.378  0.090 | no  no |
| **transported 10** | 0% | 1 | A/W | 219:Acinetobacter | **6.259** | yes |

**Supplementary File 1i: Scaled absolute abundance of ASVs shared between transported tadpoles and their adult caregivers as identified by Sourcetracker.** 16S rRNA copy numbers per microliter were determined by digital PCR (dPCR) using the QIAcuity system with the same primer set used for the v4 amplicon dataset and quantified in triplicate across different dilutions; reported values represent means with standard deviations. Relative abundances of shared ASVs, obtained from the v4 amplicon sequencing dataset, were used to scale total 16S rRNA gene copy numbers per microliter, yielding ASV-specific scaled absolute abundances. Scaled abundances corrected for the elution volume are given as copies per tadpole. Abbreviations: relA = relative abundance, absA = absolute abundance, STD = standard deviation.

| **tadpole ID** | **ASV** | **relA (%)** | **scaled absA (copies/µl)** | **scaled absA (copies/tadpole)** | **mean 16S rRNA copies/µl ± STD** |
| --- | --- | --- | --- | --- | --- |
| **transported 1** | 102:Akkermansia, 144:Enterobacteriaceae | 1.847  1.450 | 383  301 | 19141  15027 | 20727 ± 6069 |
| **transported 2** | 144:Enterobacteriaceae | 19.258 | 156 | 7804 | 810 ± 212 |
| **transported 6** | 138:Aquitalea, 421:Methylocella | 0.842  18.050 | 161  3447 | 8039  172326 | 19094 ± 458 |
| **transported 8** | 911:Staphylococcus | 10.329 | 728 | 36408 | 7050 ± 1251 |

**Supplementary File 1j: The presence of ASVs shared between transported tadpoles and their caregivers in non-transported tadpoles.**

| **tadpole ID** | **Caregiver ASVs detected in tadpole** | **ASVs** | **ASVs present in water**  **(out of 10 ASVs)** |
| --- | --- | --- | --- |
| **non-transported 1** | 1 | 144:Enterobacteriaceae | 7 |
| **non-transported 2** | 0 | 0 | 6 |
| **non-transported 3** | 0 | 0 | 7 |
| **non-transported 4** | 1 | 144:Enterobacteriaceae | 6 |
| **non-transported 5** | 1 | 144:Enterobacteriaceae | 6 |
| **non-transported 6** | 0 | 0 | 6 |
| **non-transported 7** | 1 | 144:Enterobacteriaceae | 7 |
| **non-transported 8** | 0 | 0 | 6 |
